# Supplementary material for: Cell wall and cytoskeletal contributions in single cell biomechanics of Nicotiana tabacum
Source: Quant Plant Biol. 2022 Jan 21;3:e1. doi: 10.1017/qpb.2021.15 (PMC10097588; doi:10.1017/qpb.2021.15)
Supplement: Supplementary file 1 [file S2632882821000151sup001.docx]

**SUPPLEMENTARY MATERIAL**

**Cell wall and cytoskeletal contributions in single cell biomechanics of *Nicotiana tabacum***

Leah Ginsberg^1^, Robin McDonald^1^, Qinchen Lin^2^, Rodinde Hendrickx^1^, Giada Spigolon^3^, Guruswami Ravichandran^1^, Chiara Daraio^1^, Eleftheria Roumeli^2*^

^1^ Division of Engineering and Applied Science, California Institute of Technology, Pasadena, CA 91125, USA

^2^ Department of Materials Science and Engineering, University of Washington, Seattle, WA 98195, USA

^3^ Biological Imaging Facility, California Institute of Technology, Pasadena, CA 91125, USA

* Corresponding author: eroumeli@uw.edu

*
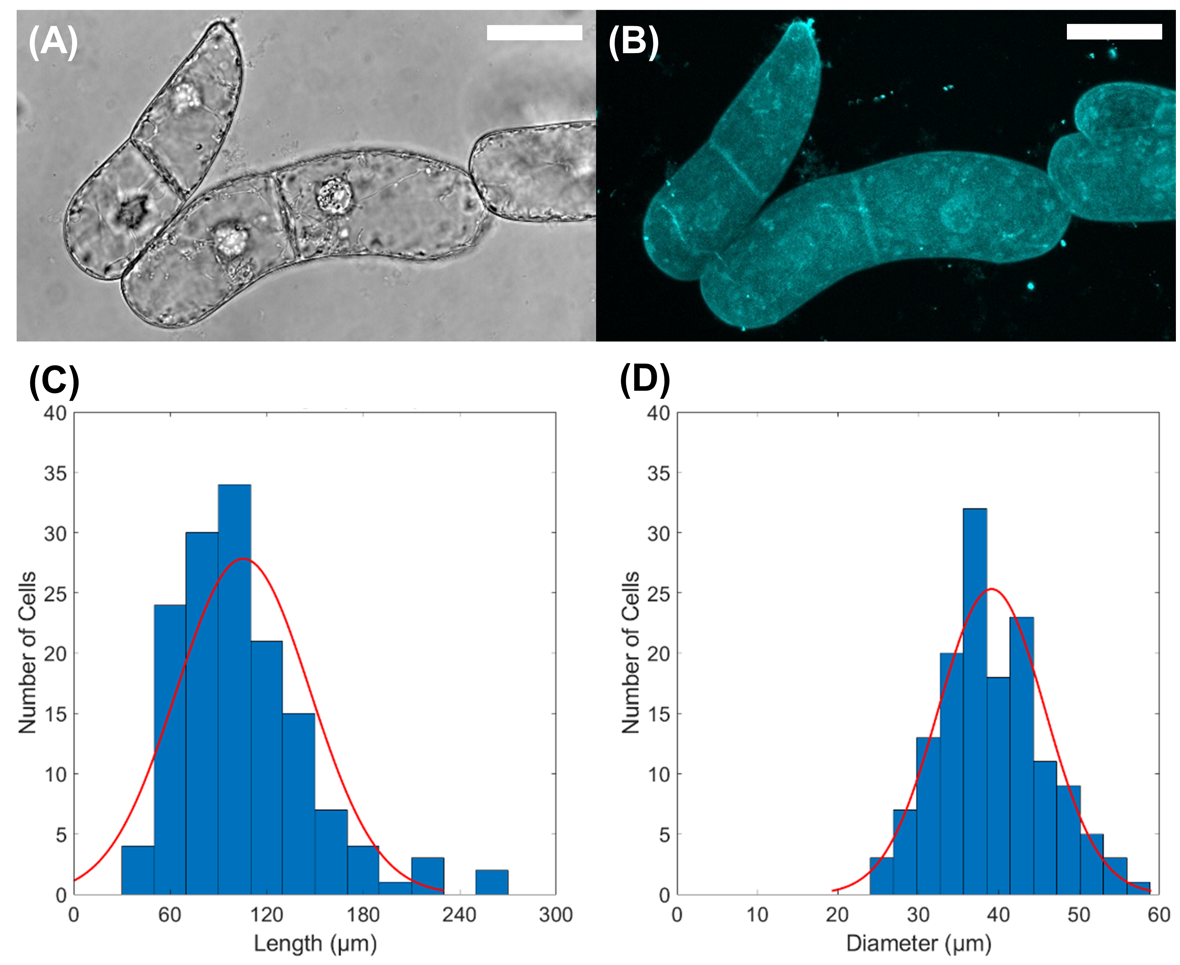
*

Figure S1. *(A) Transmitted light image from BY-2 cells under confocal microscope. (B) Fluorescent image of BY-2 cells under confocal microscope. (C) Histogram showing distribution of cell length for observed BY2 cells under confocal microscope. (D) Histogram showing distribution of cell diameter for observed BY2 cells under confocal microscope. All scale bars are 40 $\mu$m. The total number of observed cells is $n = 145$.*

*Additional Confocal Z-Stacked Images of Fluorescent Microtubules and Actin Filaments*

*
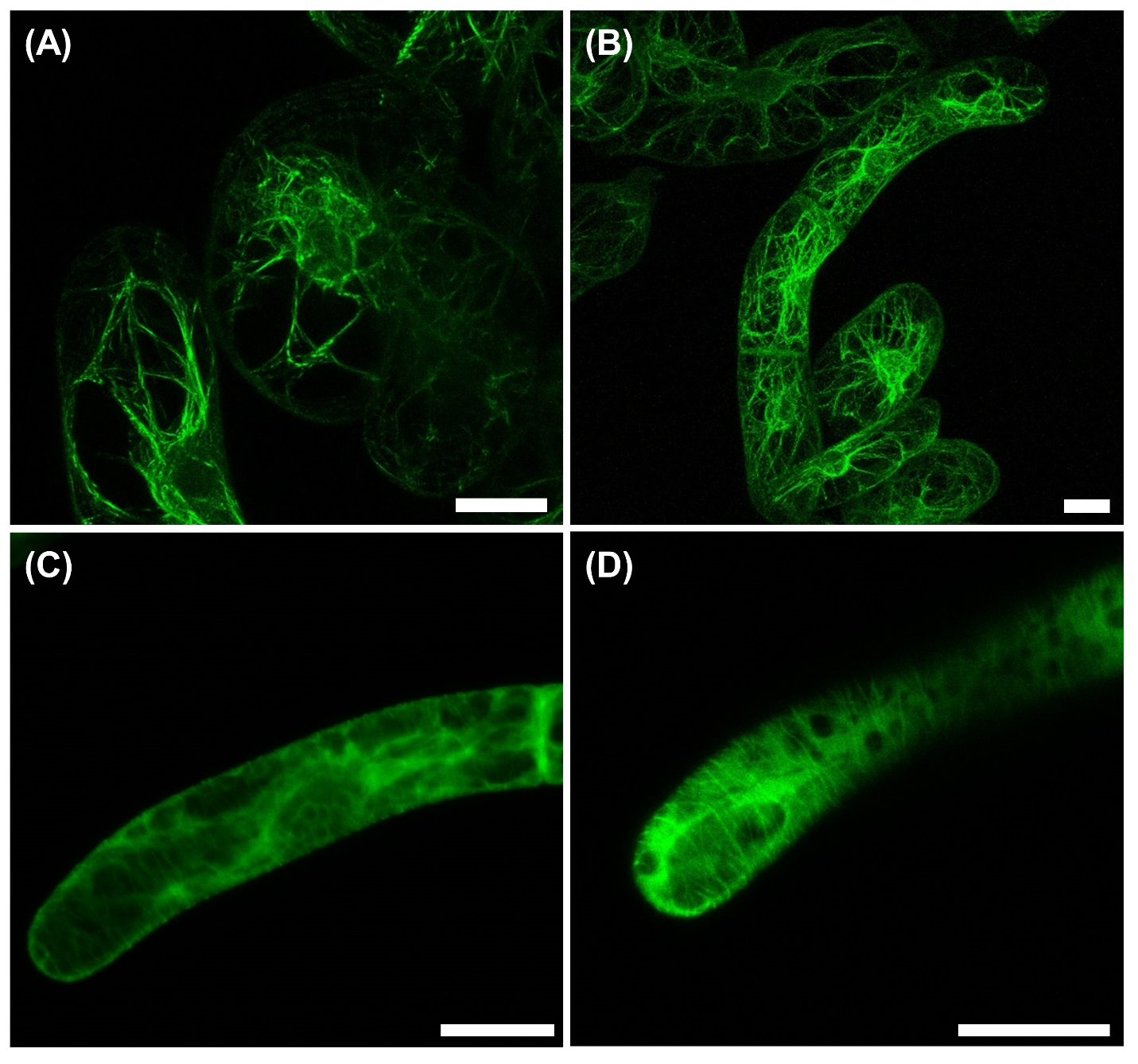
*

Figure S2. Additional confocal laser scanning microscopy (CLSM) images on BY-2 marker lines to visualize the effects of short duration drug treatments on MTs and AFs. (A) and (B) GFP-BY2-F cells in growth media-based solution, illustrating the distribution of AFs in our BY-2 cells. (C) and (D) GFP-BY2-α cells in growth media-based solution, illustrating the alignment of MTs in our BY-2 cells. Note that the MTs are oriented perpendicular to the “long” axis of the cell, mirroring the expected anisotropic orientation of cellulose in the cell wall. Images presented are Z-stacked images.

*Empirical Cumulative Distribution Functions of Stiffness and Dissipated Energy in Micro-Compression Tests*

*
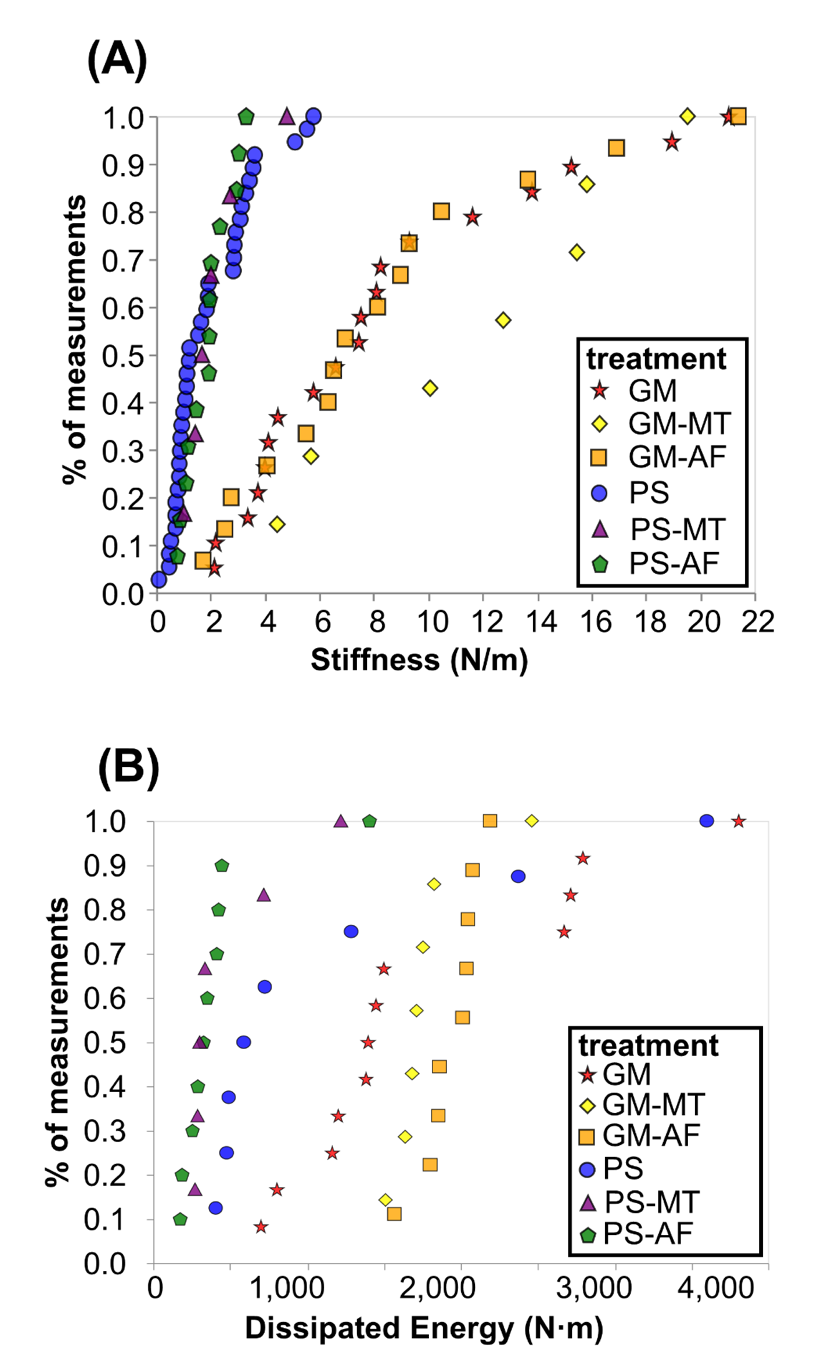
*

Figure S3. Empirical cumulative distribution functions for (A) initial cell stiffness and (B) total dissipated energy, in each test condition. Each point in the plot represents an indentation test on a different cell ($n\geq6$).

*
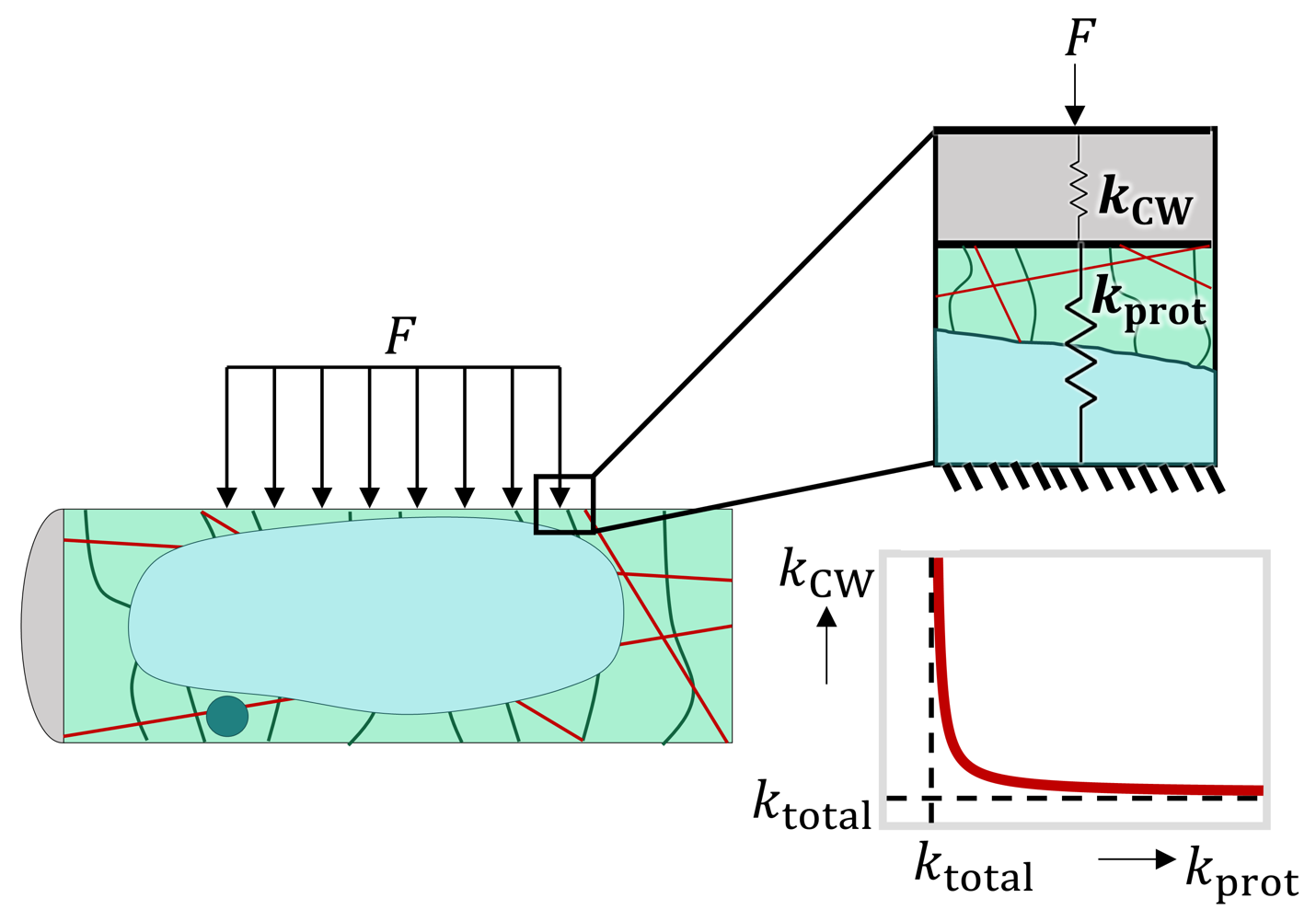
*

Figure S4. Generalized spring model for BY-2 cells in the micro-indentation test. The k_CW_ spring represents the stiffness of the CW while the k_prot_ spring represents the total stiffness of all protoplasmic components (including the vacuole, AFs (red lines), and MTs (green lines))


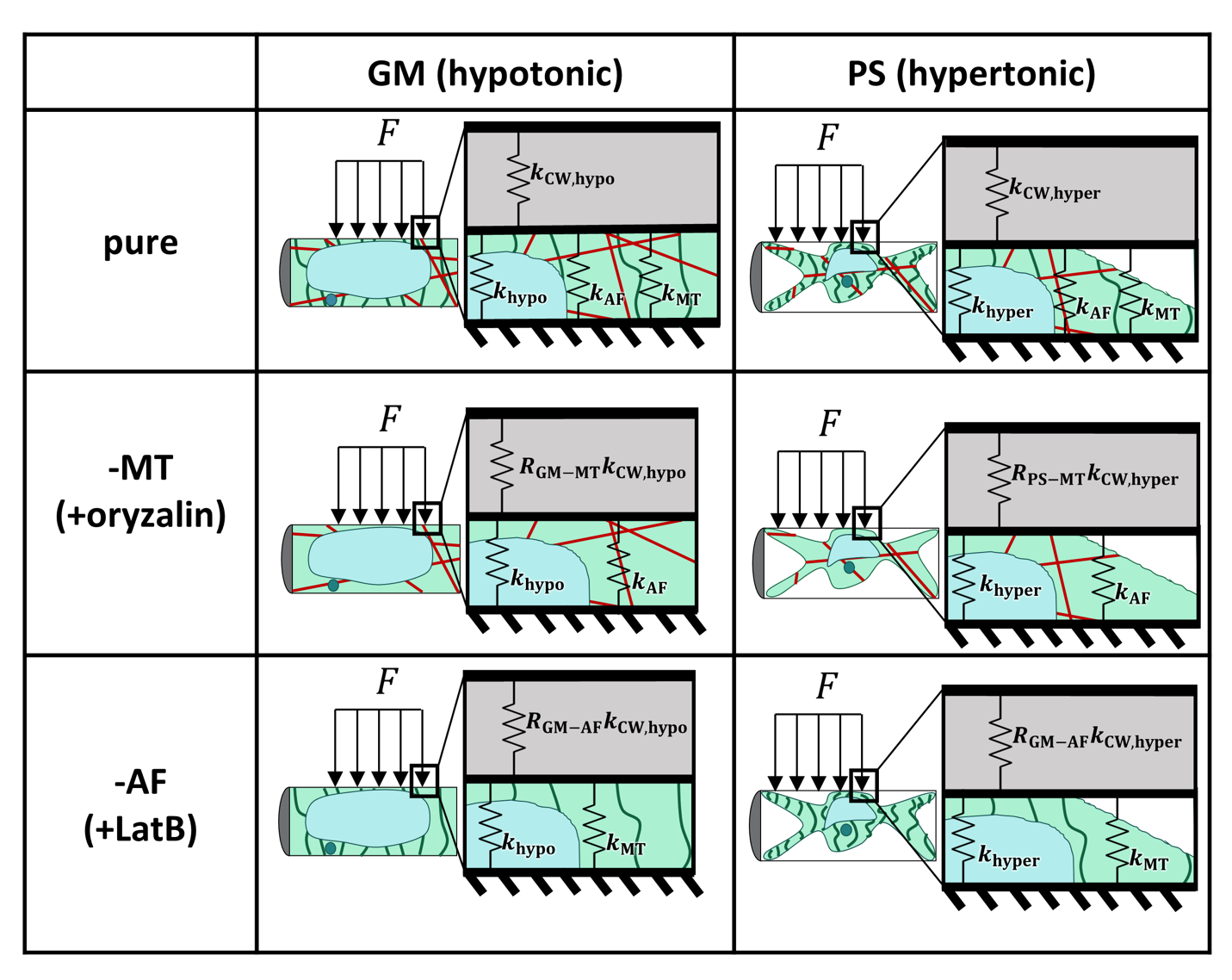


Figure S5. Table illustrating the spring model for each test condition. Left column represents cells in growth media (GM), which is a hypotonic solution for the cells, and right column represents cells in plasmolyzing solution (PS), which is a hypertonic solution for the cells. The top row represents cells with no added drug treatment. The second row represents cells with oryzalin drug treatment, which depolymerizes microtubules (MTs). The bottom row represents cells with Latrunculin B (LatB) drug treatment, which removes actin filaments (AFs).


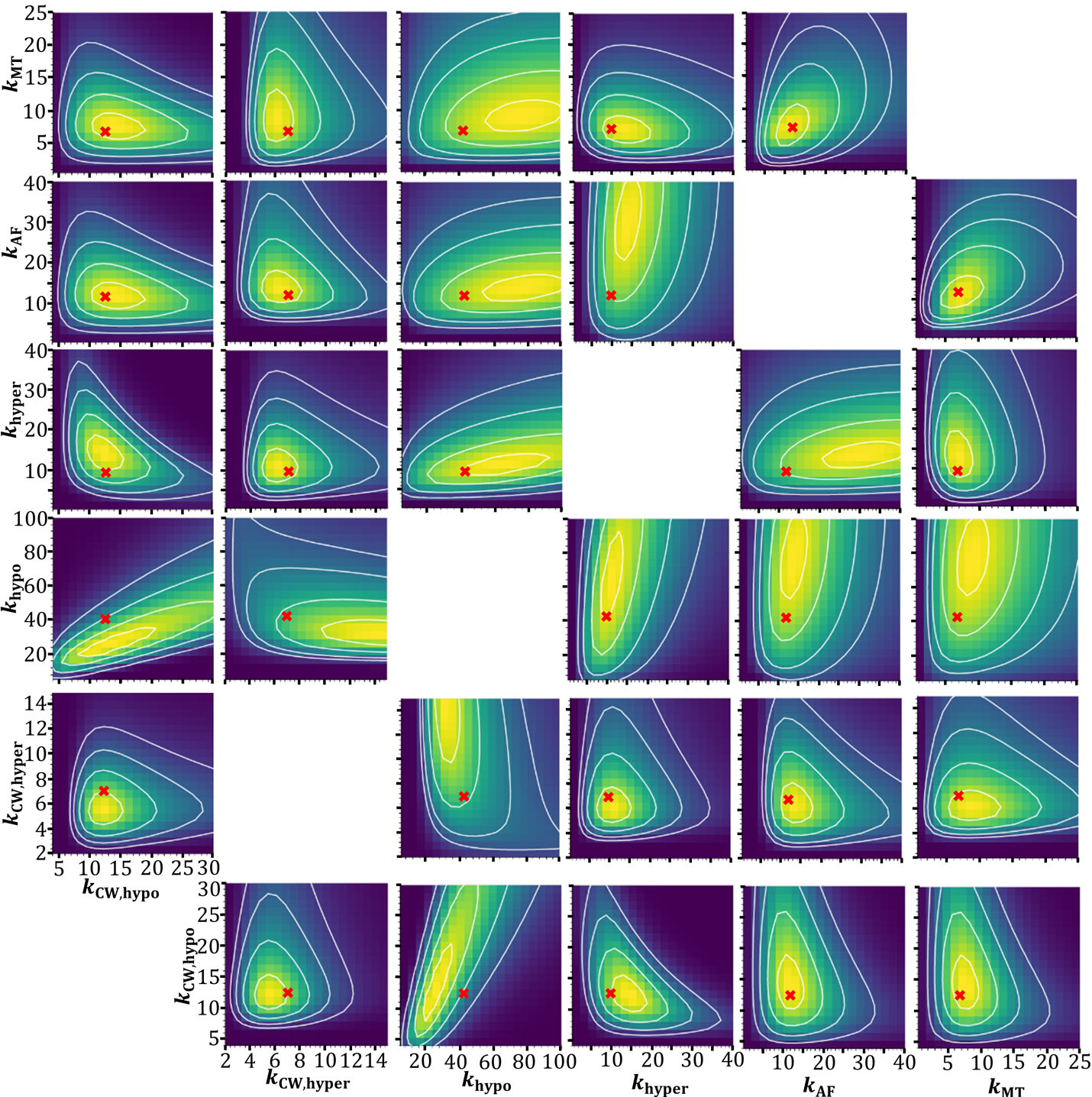


Figure S6. Contours of 6-dimensional posterior distribution projected in 2-dimensional space. Red 'x' marks the point which maximizes the posterior distribution, known as the maximum a posteriori (MAP) estimate. The white lines represent 1, 2, 3 and 4 standard deviations from the center of the distribution. All stiffness units are N/m.


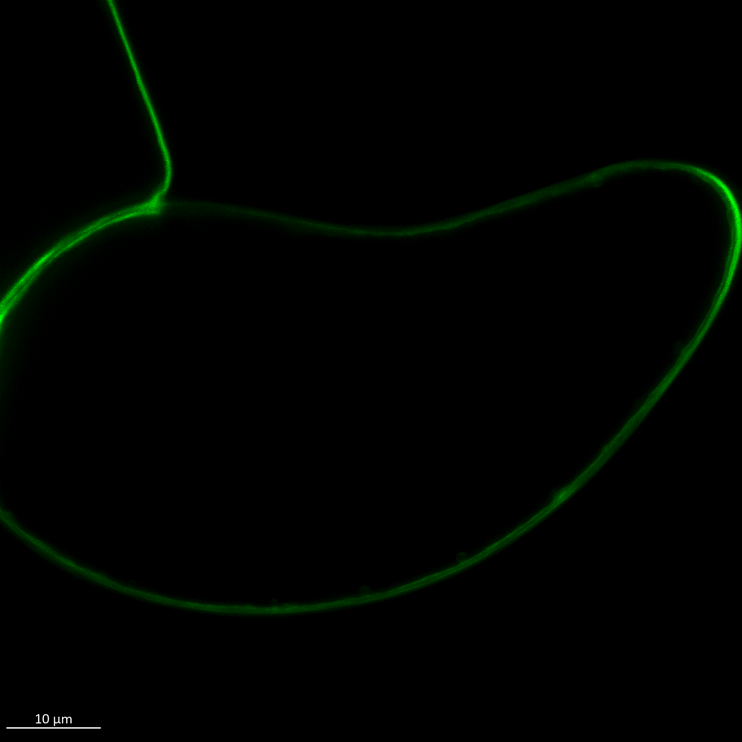


Figure S7. Representative super-resolution image of wild-type BY-2 cells stained with calcofluor white used for the CW thickness analysis.

*Bayesian Generative Model Definition*

Bayes’ theorem is:

|  | $g\left( \theta\vert y \right)=\frac{f(y\vert\theta)g(\theta)}{f(y)}$ | (2) |
| --- | --- | --- |

Note that the denominator in Bayes’ theorem, $f(y)$, called the evidence, does not depend on $\theta$, so it is a normalization constant. The evidence, $f(y)$, can be calculated:

|  | $f(y)=\int f\left( y \vert\theta\right)g\left( \theta\right)d\theta$ | (3) |
| --- | --- | --- |

where the limits of this integral are the limits of all possible values of the parameters $\theta$. It is unnecessary for our purposes to consider this term explicitly.

The central limit theorem establishes that a measurement that depends on many subprocesses tends to be Gaussian distributed. Some of the many subprocesses that affect our measurements are the variations in the effect of the drug treatment and osmolarity of the solution in which the cells are tested in, the size and shape of the cells, the orientation of the cell and subcellular components with respect to the indenter, the adhesion of the cell to the substrate and any subsequent movement of the cell under the indenter. This is not an exhaustive list but includes the most significant processes. So, we will assume that each of our sets of experimental measurements ${(k_{\mathrm{treatment}})}_{i}$ is Gaussian distributed with mean $\mu_{\mathrm{treatment}}$ and standard deviation $\sigma_{\mathrm{treatment}}$. We will also assume that each of our measurements within each treatment is independent and identically distributed. Therefore, we can define the probability density function of the likelihood for each of our measurements:

|  | $f\left( {(k_{\mathrm{treatment}})}_{i}\vert\mu_{\mathrm{treatment}}, \sigma_{\mathrm{treatment}} \right)=\frac{1}{\sqrt{2\pi{\sigma_{\mathrm{treatment}}}^{2}}}e^{-\frac{{({(k_{\mathrm{treatment}})}_{i}-\mu_{\mathrm{treatment}})}^{2}}{2{\sigma_{\mathrm{treatment}}}^{2}}}$ | (4) |
| --- | --- | --- |

Since each measurement within a specified treatment is sampled independently, the likelihood of the entire data set is a multiplication of the likelihoods for each measurement in that treatment:

|  | $f\left( k_{\mathrm{treatment}}\vert\mu_{\mathrm{treatment}}, \sigma_{\mathrm{treatment}} \right)=\prod_{i=1}^{n_{\mathrm{treatment}}} \frac{1}{\sqrt{2\pi{\sigma_{\mathrm{treatment}}}^{2}}}e^{-\frac{{({(k_{\mathrm{treatment}})}_{i}-\mu_{\mathrm{treatment}})}^{2}}{2{\sigma_{\mathrm{treatment}}}^{2}}}$ | (5) |
| --- | --- | --- |

where $n_{\mathrm{treatment}}$ is the total number of measurements in a specified treatment.

We use uninformative prior distributions for $g(\theta,\sigma$) because we have very little information about the values that these parameters will have before examining the experimental data. We know that all of the parameters must be greater than zero, and from literature on stiffness values of similar plant cells, we can anticipate that the stiffnesses will each be less than 100 N/m (Routier-Kierzkowska *et al.*, 2012; Roumeli *et al.*, 2020). By trial and error, a log-normal prior distribution for the parameters $\theta$ was found to cover the range of expected values and give reasonable resulting data sets:

|  | $g(\theta)=\frac{1}{0.75*\theta\sqrt{2\pi}}e^{-\frac{{(\ln x-\ln15)}^{2}}{2*{0.75}^{2}}}$ | (6) |
| --- | --- | --- |

The standard deviation in each treatment, $\sigma$, is assumed to be proportional to the magnitude of the mean stiffness in each treatment:

|  | $\sigma_{\mathrm{treatment}}=0.5*\mu_{\mathrm{treatment}}$ | (7) |
| --- | --- | --- |

Finally, we assume that the distributions for $\theta$ and $\sigma$ are independent so that:

|  | $g\left( \theta,\sigma\right)=g\left( \theta\right)g(\sigma)$ | (8) |
| --- | --- | --- |

With the prior distributions defined, we can generate data sets from the fully generative model. These generated data sets are known as prior predictive checks, and they ensure that the model does not generate many unrealistic or unphysical values when drawing random data sets from the previously defined distributions. See Figure S1 for example data sets drawn from the completed generative model.


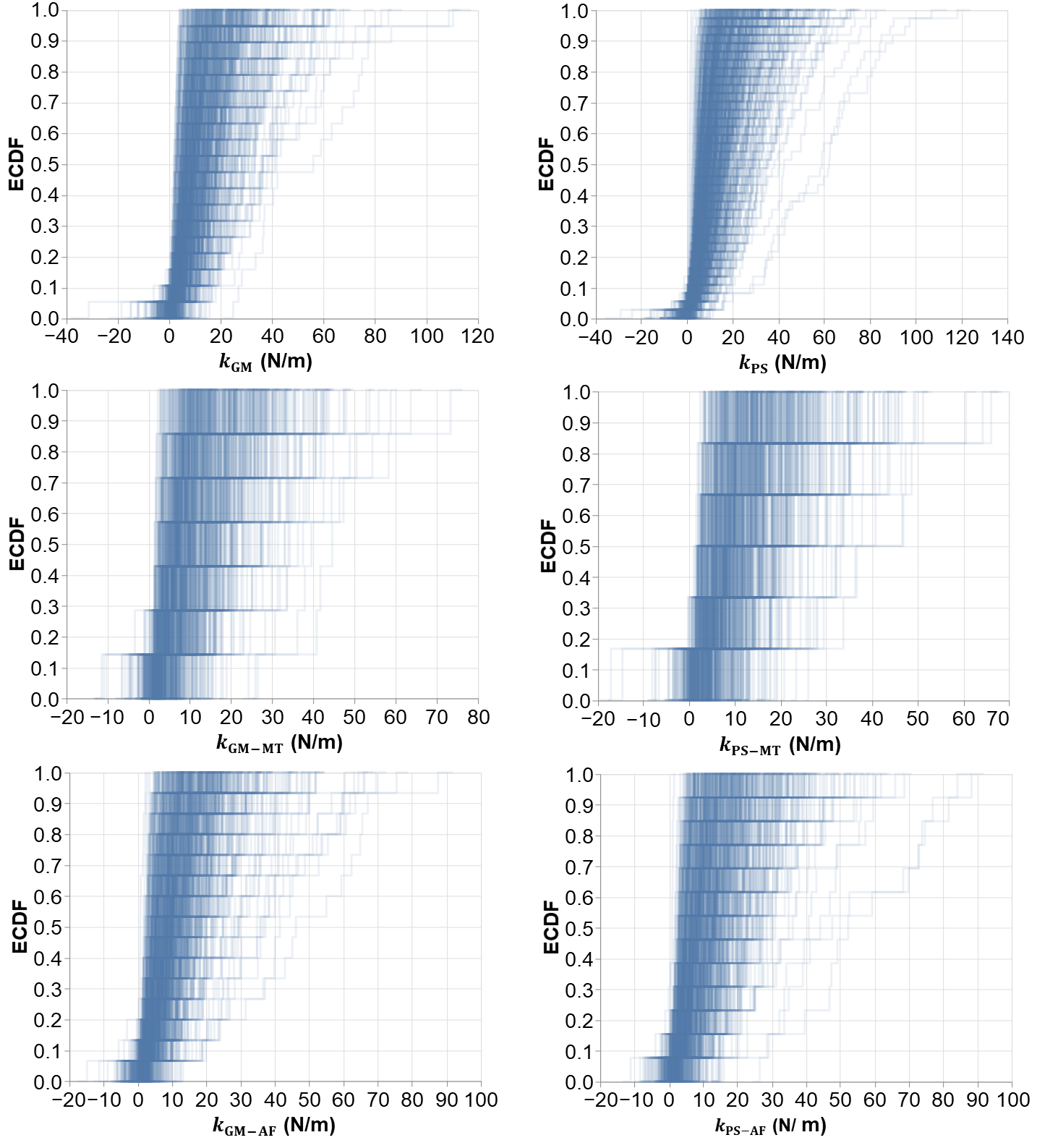


Figure S8. Prior predictive checks. Each line represents a sample data set drawn using the fully generative model described in equations above. Each subplot contains 500 draws of data sets that are the same size as the respective number of experimental samples in that treatment.
